# When Professional Boundaries Are Crossed: Reflections on Stalking and Clinician Safety in Psychiatric Training

**DOI:** 10.1192/bjo.2026.11871

**Published:** 2026-06-30

**Authors:** Sophie Nocton, Lian Chua, Sara Mansoor, Sarfaraz Shora

**Affiliations:** 1 Leeds Community Healthcare NHS Trust, Leeds, United Kingdom; 2 Bradford District Care NHS Foundation Trust, Bradford, United Kingdom

## Abstract

**Aims:**

Stalking is an under-recognised occupational hazard within mental health services and disproportionately affects women clinicians. Boundary violations by current or former patients can pose risks to clinician safety, disrupt professional identity, and generate significant emotional distress. Experiences of stalking are often under-reported, and clinicians may feel uncertain about how best to seek support.

This case study reflects on the professional, organisational and emotional learning arising from a single experience of stalking encountered by a female psychiatrist during her Core Psychiatry training in Yorkshire, UK. The hypothesis underpinning this work is that, for this clinician, early institutional support, clear boundary-setting and access to protected reflective spaces mitigated psychological harm and supported resilience.

**Methods:**

The case involved escalating stalking behaviour from a former patient, targeted towards a female Core Psychiatry trainee. This progressed from letters and unsolicited gifts to persistent social media contact, culminating in messages containing threats of harm and suicide. As a result, the clinician experienced fear, hypervigilance and moral tension between compassion for a mentally unwell patient and the need to preserve personal safety. The experience was explored using a narrative-reflective approach and supported through clinical and educational supervision, Balint group discussion, police involvement, advice from specialist stalking services, and implementation of digital-safety measures.

**Results:**

Reflection on this case identified learning points specific to the author’s experience. Early reporting reduced isolation and enabled coordinated risk management. Clear documentation supported organisational responses and escalation. Proactive digital-identity management increased perceived safety. Explicit acknowledgement of gendered vulnerability was validating and reduced self-blame. Supervision and Balint groups provided emotional containment and helped reframe vulnerability as professionalism rather than weakness. For this clinician, maintaining clear boundaries supported wellbeing without undermining compassionate patient care.

**Conclusion:**

This reflective case highlights how stalking may affect clinician safety, professional identity and emotional wellbeing within psychiatric training. While experiences and responses will vary, this account suggests that organisational openness, supervisory support and access to reflective spaces were helpful in this instance. Sharing individual experiences may support awareness and encourage dialogue about clinician safety and boundary-violating behaviour within mental health services.

